# Introgression of domesticated salmon changes life history and phenology of a wild salmon population

**DOI:** 10.1111/eva.13375

**Published:** 2022-04-11

**Authors:** Francois Besnier, Fernando Ayllon, Øystein Skaala, Monica Favnebøe Solberg, Per Tommy Fjeldheim, Kaja Anderson, Sofie Knutar, Kevin Alan Glover

**Affiliations:** ^1^ 115347 Institute of Marine Research Bergen Norway; ^2^ Department of Biological Sciences University of Bergen Bergen Norway

**Keywords:** Atlantic salmon, feralization, genetic introgression

## Abstract

The release of domesticated conspecifics into the natural environment, whether deliberate or accidental, has the potential to alter the genetic integrity and evolutionary trajectory of wild populations. This widespread challenge is of particular concern for wild Atlantic salmon. By investigating phenotypic differences between the offspring of domesticated, hybrid, and wild Atlantic salmon released into the natural environment, earlier studies have documented the short‐term consequences of introgression from domesticated fish into wild salmon populations. However, few studies have investigated the joined product of introgression and natural selection after several generations. Here, we investigated the phenotypic response of an Atlantic salmon population that has been subjected to an average of 24% genetic admixture by domesticated conspecifics escaping from fish farms over three decades (approximately 6–7 generations). Individual levels of admixture were positively correlated with increased size at the smolt and adult stages for both sexes, a decrease in the age of male smolts, and a decrease in the age at maturity for males. These life history changes are presumably the consequence of the well‐documented directional selection for increased growth in domesticated salmon and are likely maladaptive. However, the most novel result of this study is that admixture was positively linked with delayed date of return to the river, with highly admixed fish arriving up to 26 days later than nonadmixed fish. Potentially, this phenological change provides admixed individuals with a survival advantage in the later phase of the life cycle as it reduces their period of exposure to selection through rod and line angling. We, therefore, conclude that while gene flow from domesticated conspecifics changes life history and phenological traits of wild Atlantic salmon populations, most of which are likely to be maladaptive, when pressured by additional anthropogenic challenges, some changes may confer a fitness advantage for a short part of the life cycle.

## 1 INTRODUCTION

Domestication and selective breeding underpins all modern agri‐ and aquaculture (Gupta, 2004; Teletchea & Fontaine, 2014). In the human‐controlled environment, domesticated strains outperform their wild conspecifics in the trait(s) under directional selection, thus increasing production and profitability. In the natural environment, however, the performance of domesticated, hybrid, and admixed individuals is typically lower than for their wild conspecifics. In addition, they often display phenotypic differences that are likely maladaptive (Fleming et al., 2000; Gering et al., 2019; Julian, 1998; McGinnity et al., 2003; Price, 1999; Skaala et al., 2012). Therefore, the release of domesticated organisms into the natural environment, whether it be deliberate for population augmentation or accidental via escape, is of widespread concern.

Introgression of domesticated conspecifics in wild populations occurs in a wide range of species and habitats around the world (Laikre et al., 2010), including examples such as fish (Glover et al., 2013; Karlsson et al., 2016), wolves (Verardi et al., 2006), mink (Kidd et al., 2009), wild boars (Goedbloed et al., 2013), and cats (Lecis et al., 2006). Looking specifically at the family Salmonidae, which includes several species of trout and salmons, many populations have been exposed to introgression and admixture from nonlocal and sometimes domesticated conspecifics from a wide variety of sources. This includes examples such as deliberate population augmentation using local wild broodstock (Hess et al., 2012; Letourneau et al., 2018), deliberate population augmentation using nonlocal and/or captive‐reared, and therefore inadvertently domesticated broodstock (Hansen et al., 2009) and accidental supplementation by nonlocal domesticated fish that have also been subjected to directional selection for production‐related traits in commercial aquaculture (Glover et al., 2013; Karlsson et al., 2016; Wringe et al., 2018). As a consequence of these extensive activities, there is a large body of literature examining the loss in fitness of salmonid fishes released from hatcheries and fish farms into the wild (Araki et al., 2008; McGinnity et al., 2003; Milot et al., 2013), in addition to the effects that introgression and admixture from these released fish has on wild populations (Bolstad et al., 2017; Muhlfeld et al., 2009; Naish et al., 2007). However, one of the best examples of introgression and admixture, that is, that originating from nonlocal and highly domesticated fish subjected to directional selection for production‐related traits of importance in commercial aquaculture, the Atlantic salmon (*Salmo salar*) arguably serves the best example from the family Salmonidae.

Atlantic salmon displays a history of domestication spanning half a century, where extensive escapes from aquaculture have left a globally unprecedented legacy of impacts on wild salmon populations (Glover et al., 2017). Domesticated Atlantic salmon that have escaped from fish farms have been observed in wild populations for several decades (Diserud, Fiske, et al., 2019; Glover et al., 2019; Morris et al., 2008; Walker et al., 2006), and widespread introgression is documented (Glover et al., 2013; Palm et al., 2021; Karlsson et al., 2016; Wringe et al., 2018). Domesticated Atlantic salmon have been selected for a range of economically important production traits for approximately 15 generations since the early 1970s, including examples such as fast growth, delayed maturation, disease resistance, and survival (Gjedrem, 2000, 2010). As a result of this selection regime, relaxed natural selection, and general adaptation to the aquaculture environment, domesticated Atlantic salmon now display a wide range of genetic differences to their wild conspecifics (Glover et al., 2017; Naval‐Sanchez et al., 2020). In common garden experiments conducted in the farming environment or semi‐natural conditions, and in comparison with their wild conspecifics, domesticated Atlantic salmon display faster growth (Glover et al., 2009; Solberg et al., 2013), reduced precocious parr maturation rates (Harvey et al., 2018), altered gene‐transcription profiles (Bicskei et al., 2014; Roberge et al., 2006), higher stress tolerance (Solberg et al., 2013), reduced predator awareness (Debes & Hutchings, 2014; Houde et al., 2010), and increased susceptibility to predation (Solberg et al., 2020).

Common garden studies conducted in the natural environment have demonstrated that the offspring of domesticated Atlantic salmon display reduced survival in freshwater and saltwater, in addition to greater size at age and lower smolt age (Besnier et al., 2015; Fleming et al., 2000; McGinnity et al., 1997, 2003; Skaala et al., 2012, 2019). Furthermore, common garden studies have revealed differences in phenology, specifically the seasonal timing of smolt migration (Skaala et al., 2019). These pioneering common garden experiments have more recently been complimented by a small but growing number of studies looking at the response of Atlantic salmon populations subjected to gene flow from domesticated fish escaping from fish farms, either from single events or cumulative introgression. Concurring with the results of common garden studies, these more recent studies of natural populations displaying admixture have also demonstrated poorer survival of domesticated, hybrid, or admixed fish (Sylvester et al., 2019; Wacker et al., 2021; Wringe et al., 2018), as well as changes in the life history traits age and size at adult maturation (Bolstad et al., 2017, 2021). Nevertheless, although this growing literature contributes to our understanding of the consequences of introgression of domesticated conspecifics in wild Atlantic salmon populations, there is still a need for further studies to elucidate and quantify the full magnitude of this challenge.

**FIGURE 1:**
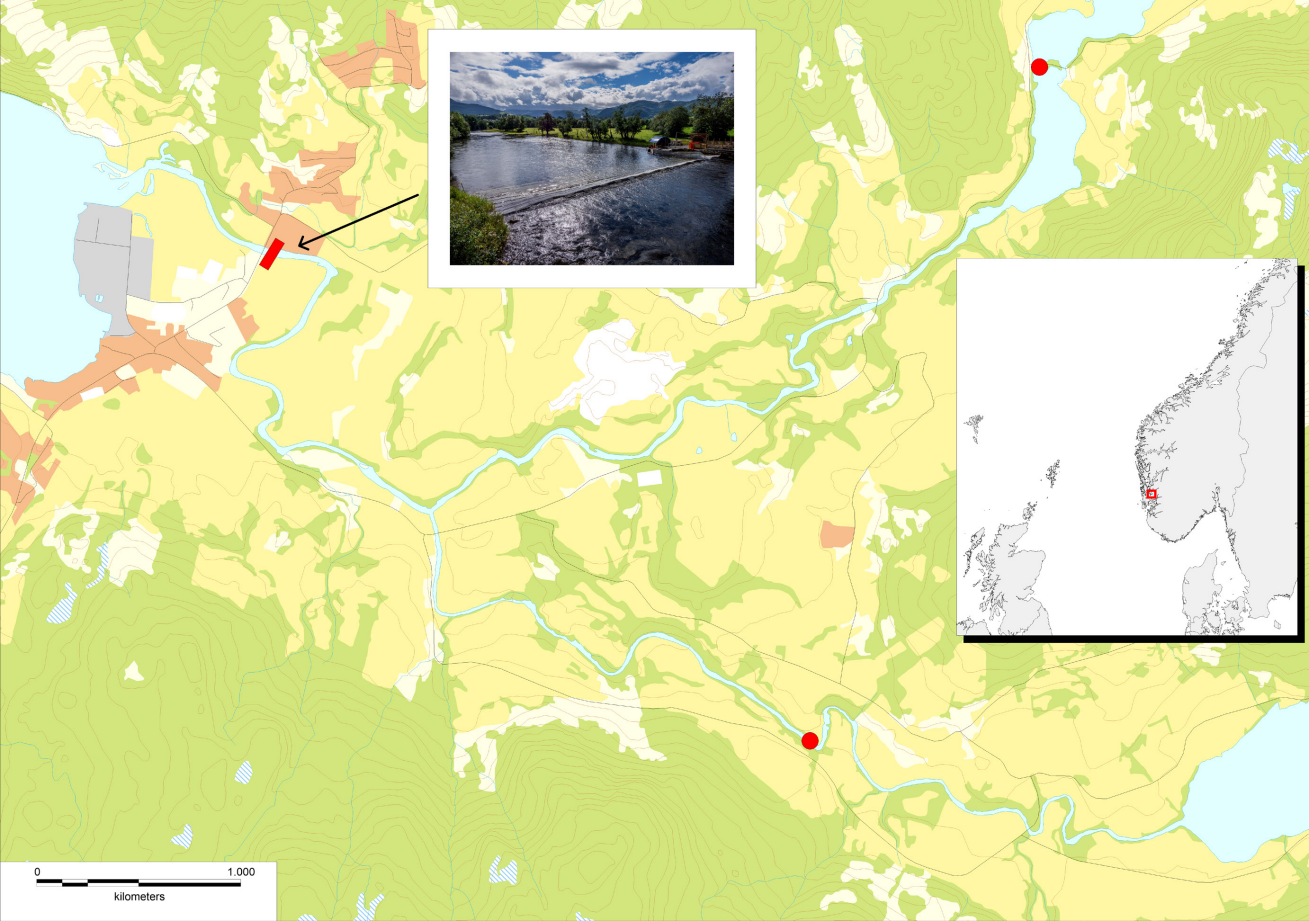
Location of the river Etneelva and insert picture of the upstream migration trap where adults entering the river are sampled

In 2013, and in response to high proportions of domesticated farmed Atlantic salmon escapees entering the river yearly since the late 1980s (Diserud, Fiske, et al., 2019; Glover et al., 2019), an upstream fish trap was established in the river Etne western Norway (Figure 1). This trap permits sampling the adult population returning to the river, and simultaneously, allows for removal of farmed escapees to mitigate further introgression. As a result of introgression from domesticated escapees entering the river in the period from the late 1980s to 2012, the population inhabiting the river now displays an average of approximately 20% admixture (Glover et al., 2013; Karlsson et al., 2016). Despite this relatively high degree of admixture, the adult population entering the river each year is typically in the range of 1000–2000 individuals (Harvey et al., 2017). In turn, this means that the evolutionary trajectory of this admixed population is not likely to be strongly influenced by the stochastic effects of genetic drift, thus making selection and gene flow the primary drivers of temporal genetic changes. Therefore, and when seen in context of the upstream fish trap that permits sampling the adult population, this river provides a novel system in which to study the long‐term effects of admixture from domesticated Atlantic salmon. The present study utilized this opportunity to investigate the effects of admixture on a range of phenotypic traits in the wild population. To achieve this, we first used a panel of 50,000 SNPs to identify markers to compute individual admixture, and thereafter, we compared the individual admixture estimates with a range of phenotypic and phenological measurements of the population.

## 2 MATERIALS AND METHODS

### 2.1 Samples

The study is based upon the following three sets of samples. (1) 797 wild adult salmon captured by angling in the river in year 1983/84. These represent the historical baseline sample, that is, preaquaculture impact, all of which are hatched in the wild and are of 100% wild ancestry. (2) 350 domesticated farmed salmon escapees removed from the river in the period 1989–2012 (15 per year). These fish represent a random sample of the farmed escapees that have introgressed in the wild population. They originate from multiple commercial strains that were collectively founded upon fish from over 40 Norwegian rivers in the early 1970s (Gjedrem et al., 1991). (3) 751 wild adult salmon were captured in the upstream fish trap in the years 2013 to 2016. These fish represent the contemporary samples for the wild population, that is, postaquaculture impact. All the fish in the contemporary sample were hatched in the wild and display variable degrees of admixture computed on a scale between 0 (i.e., pure wild ancestry) and 1 (i.e., pure domesticated ancestry) (see methods below for computation of admixture). For simplicity and consistency throughout the paper, we refer to these fish as nonadmixed, admixed, and fully admixed. The historical wild samples and the samples of the farmed escapees were used for genetic analyses to identify markers to compute individual admixture in the contemporary sample, while the contemporary samples were used to investigate phenotypic changes associated with their individual computed admixture scores.

All fish included in this study, that is, both wild and domesticated, had their scales read prior to genetic analysis in order to verify their origin, that is, hatched in the wild or in captivity. This was conducted according to the method described by Lund and Hansel (1991) and as implemented in the national monitoring program for farmed escapees in more than 220 Norwegian rivers annually (Glover et al., 2020). For the contemporary samples, the following biological information was also stripped from their fish scales following the method described by Lea and Dahl (Dahl, 1910; Lea, 1910): smolt age and back‐calculated smolt size, age at maturation and back‐calculated growth to the first annual zone (size after first winter at sea). In addition, the size (length and weight) and date of fish captured in the upstream fish trap were recorded manually on calibrated scales and a measuring table prior to releasing them back into the river to continue their upstream migration.

Historical wild samples from angling were collected by biologist Erlend Waatevik and donated to Øystein Skaala for scientific studies. The current study was initiated and conducted in close collaboration with the Hordaland County Governor, the Norwegian Environment Agency and the Norwegian Directorate of Fisheries. All contemporary samples were taken from the trap with the following research permit from the Norwegian animal experimentation body (permitID2416, ID 11798, and ID19355), and all scales of farmed escapees were provided by the Norwegian Institute for Nature Research (NINA), after being collected by the local angling club that removed escapees from the river over the 2–3 decade period prior to installation of the fish trap.

### 2.2 Genetic markers to compute domestication‐admixture

For all samples, DNA was isolated using Qiagen DNAeasy blood and tissue kits in 96‐well format. Thereafter, samples were genotyped on a ThermoFisher Axiom 57K single‐nucleotide polymorphism (SNP) array (NOFSAL03, 55735 markers) that was developed by Nofima (Norwegian institute for applied research in food aquaculture and fisheries) in collaboration with private aquaculture companies Mowi and SalmoBreed. SNP markers were quality checked following the “Best Practice Workflow” on the Affymetrix axiom analysis software (available at: https://www.thermofisher.com/no/en/home/technical‐resources/software‐downloads.html). SNPs with call rates lower than 0.97 and samples with call rates lower than 0.85 were discarded, whereas markers classified as “PolyHighResolution” (high resolution in both homozygous and heterozygous clusters) were conserved for further data analysis.

Using the genotypes from PolyHighResolution SNPs, the genetic distance (F_ST_) was computed between historical wild and domesticated escapees for each SNP using the R package hierfstat (Weir & Goudet, 2017). As an alternative method to identify SNPs segregating historical wild and domesticated escaped salmon, we also performed a Discriminant Analysis of Principal Components (DAPC) (Jombart et al., 2010) (Figure S1). The variance contribution of each SNP on the discriminant axis was then compared with the computed F_ST_ value for each individual locus. Three marker subsets were then selected by retaining SNPs that provided simultaneously the highest 1%, 5%, or 20% F_ST_ and discriminant variance contributions. These new reduced panels of markers were expected to contain the relevant genetic information to differentiate historical wild from domesticated salmon samples while containing a smaller number of SNPs, thus permitting computation of individual admixture without very excessive run times (see below).

### 2.3 Genetic sex determination

Genetic sex was identified by the presence of the sdY gene (Eisbrenner et al., 2014; Yano et al., 2012); if the presence of exon 2 and 4 were detected, the individual was designated as male. An ABI Applied Biosystems ABI 3730 Genetic Analyser was used for fragment analysis, the outputs of which were used to call genotypes in GeneMapper (Applied Biosystems, v. 4.0). This assay gives accurate identification of sex, although a very low percentage of fish identified as genetic males are phenotypic females due to carrying an inactive pseudo‐copy of the sdY gene with both exon 2 and 4 (Ayllon et al., 2020).

### 2.4 Computation of individual admixture

The three SNP subsets were used to estimate individual admixture in the contemporary sample using the individual assignment program STRUCTURE (Pritchard et al., 2000), with 10,000 burn‐in and 100,000 repetitions. We followed a standardized method for detecting unilateral gene flow (Karlsson et al., 2014), where the admixture of each individual was computed, in turn, in a separate STRUCTURE run. Each STRUCTURE run involved one test individual without a prior ancestry provided to the program, together with 250 historical wild and 250 domesticated reference individuals with known ancestry. The procedure was performed in the R package ParallelStructure (Besnier & Glover, 2013) using 20 CPUs on a Linux centos7 server with 36 cores and 512 GB of random access memory available.

The individual assignment from STRUCTURE provided the probability of belonging to the wild reference population *p*(wild), for each tested individual from the contemporary sample. To calibrate the average admixture estimates, a subset of 100 individuals from the historical wild samples and 100 individuals from the domesticated samples were also tested without an ancestry prior. From this, we computed the average *p*(wild) for the wild historical (Wild_ref_) and an average *p*(wild) for the domesticated population (Farm_ref_). Because wild_ref_ was smaller than one, and Farm_ref_ larger than zero, the average admixture of the contemporary population was calibrated as follows:

Proportion of wild genome = $\frac{\bar{p(\mathrm{Wild})}‐{\mathrm{Farm}}_{\mathrm{ref}}}{{\mathrm{Wild}}_{\mathrm{ref}}‐{\mathrm{Farm}}_{\mathrm{ref}}}$ (Karlsson et al., 2014).

Where $\bar{p(\mathrm{Wild})}$ is the average *p*(wild) of the contemporary samples, and 1−*p*(Wild) is the resulting estimate of individual admixture used to compute the correlation between admixture and phenotypes.

### 2.5 Testing admixture estimation accuracy

To test the accuracy of admixture estimation from the three SNP subsets, a set of hybrid individuals was generated by simulating crosses between the available wild historical and domestic escaped samples. A total of 200 F1 hybrids were simulated using the *hybridize* function from the ADEgenet R package (Jombart, 2008). The expected admixture level of the hybrid individuals (0.5) was compared with the estimated raw admixture *p*(wild) obtained as described above.

### 2.6 Statistical analyses

After estimating individual admixture for each salmon in the contemporary sample, we computed the correlation between the raw admixture estimate *p*(wild), and size (length and weight) upon capture in the fish trap, sea age and date of entry to the fish trap. Smolt age, smolt size and size at first sea winter, both deduced from scales, were also considered.

The correlation between body size and admixture was estimated by linear model:

Y=SW+sex+admix+year+e

Where Y is the measured phenotype, SW is the number of winters at sea, sex is a binary variable for genetically determined sex, admix is the estimated genetic admixture, year factor is accounting for the capture year, and e is a vector of normally distributed residuals.

Similar to fish size, day of capture was recorded, and the correlation between admixture and phenotype was estimated by linear model including the same covariates.

For back‐calculated length at first sea winter and back‐calculated smolt length, a similar model was used without sea age covariate.

The age at which each fish started smolt migration (from river to sea) was inferred by scale reading. As 99% of the fish started smolt migration as 2 year old or 3 three year old, smolt age was modeled as a binary trait, and the correlation between the probability of migrating to sea as a 3‐year‐old smolt and admixture was estimated by a generalized linear model of the binomial family and a logit link function:

$$\log\left(\frac{p_{\left(3\right)}}{1‐p_{\left(3\right)}}\right)=\text{admix}*\text{sex}+\text{year}+e$$

Where *p*_(3)_ is the probability to migrate as a 3‐year‐old smolt, sex is a binary variable for genetically determined sex, admix is the estimated genetic admixture, and e is a vector of normally distributed residuals.

Similar to smolt age, sea age was modeled as a binary trait consisting of early maturing fish (one sea winter) against late maturing fish (2+ sea winter). The correlation between the probability of staying one winter and admixture was estimated by a generalized linear model of the binomial family and a logit link function.

$$\log\left(\frac{p_{\left(1\right)}}{1‐p_{\left(1\right)}}\right)=\text{admix}*\text{sex}+\text{year}+e$$

Where *p*_(1)_ is the probability to stay 1 winter in the sea.

## 3 RESULTS

Due to lower DNA quality of the historical samples, nearly 50% of the SNPs displayed a call rate value below the recommended threshold (0.97). The remaining 50% SNPs fulfilled the criterion recommended by the Axiom Analysis software. After quality checking, this approach resulted in 22,571 PolyHighResolution SNPs that were used in the next step of the analysis.

**FIGURE 2:**
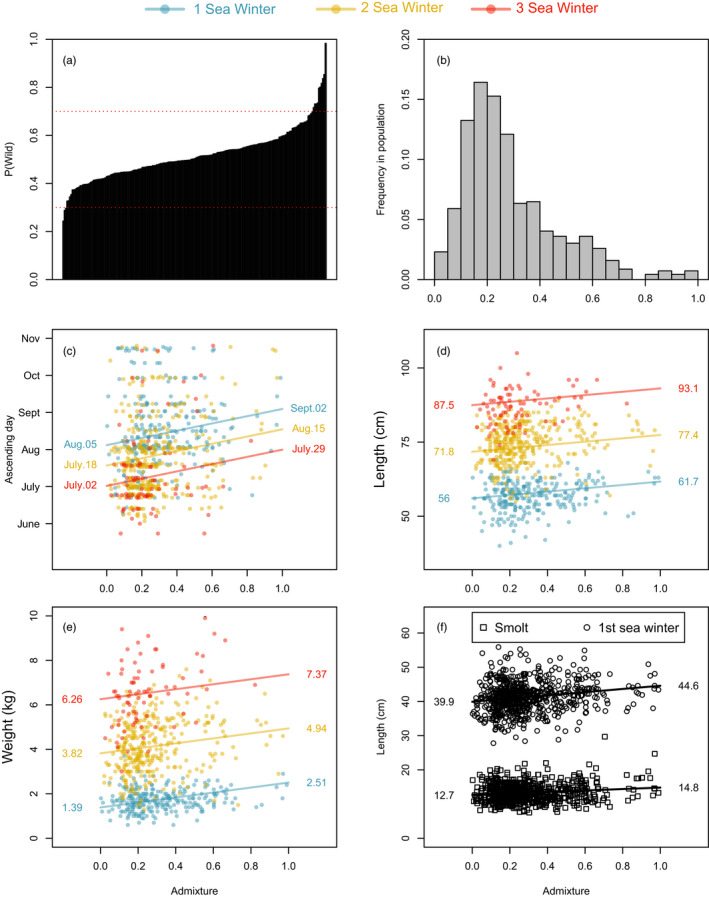
Individual admixture level estimated in the simulated F1 hybrids (a), distribution of estimated admixture in the contemporary population (b), admixture plotted against ascending day (c), length upon entry to the river (d), weight upon entry to the river (e), and back‐calculated length at smolt and during the first winter at sea (f)

The three marker subsets that included SNPs providing the highest 1%, 5%, and 20% F_ST_ and variance contribution values consisted of 142, 494, and 2670 markers, respectively. When testing the accuracy of admixture estimates in the simulated hybrids, accuracy increased as expected with the number of markers. The mean squared difference between expected and estimated admixture was equal to 0.017, 0.013, and 0.011 in the 1%, 5%, and 20% SNP subsets, respectively. Furthermore, 95% of the estimated admixture values for the simulated hybrids were included in the interval 0.20–0.8 for the 1% panel, 0.27–0.73 for the 5%, and 0.30–0.70 for the 20% panel (Figure 2a). Computation times were also increasingly long with the number of markers. Computing admixture for all the samples required *circa* 2 days for the 1% SNP subset and *circa* 20 days for the 20% subset.

Using the most accurate subset of SNPs, that is, the set of 2670 SNPs, the adjusted mean level of admixture was computed at 24% in the contemporary sample, with 9% of the population displaying admixture lower than 10%, and 58% of the population displaying between 10% and 30% admixture (Figure 2b).

A significant effect of admixture was detected on size at age (increase, both sexes, freshwater and marine), smolt age (decrease, males only), date upon entry to the fish trap across age groups (increase, both sexes), and age at maturation (decrease, males only). The date upon return to the river, that is, entry to the fish trap, was positively correlated with admixture for both males and females (*df* = 1, *F* = 18.0, *p* = 2.0 × 10^−5^). As individual admixture varied on a scale of 0–1, the model prediction estimated that on average, fully admixed fish migrated into the river 26 days later than nonadmixed fish, with an average migrating date *circa* July 16 for nonadmixed fish, *circa* July 27 for 0.5 admixed fish, and *circa* August 10 for the fully admixed fish (Figure 2c).

Within each sea age group, adult fish size (i.e., size upon return to the river) was positively correlated with admixture for both length (*df* = 1, *F* = 16.2, *p* = 5.9 × 10^−5^) and weight (*df* = 1, *F* = 23.9, *p* = 1.2 × 10^−6^). Based on model predicted values, fully admixed fish were on average 5.7 ± 1.3 cm longer and 1.2 ± 0.24 kg heavier than nonadmixed fish (Figure 2d,e). The effect of admixture was stronger on females than on males, with fully admixed males being on average 3.8 ± 1.8 cm longer (0.7 ± 0.3 kg heavier) than nonadmixed males and fully admixed females being on average 8.3 ± 1.9 cm longer (1.9 ± 0.39 kg heavier) than nonadmixed females. Similarly, the back‐calculated length at smolt (*df* = 1, *F* = 14.5, *p* = 1.5 × 10^−4^) and length after ~7 months in the sea (*df* = 1, *F* = 30.3, *p* = 4.9 × 10^−8^) were positively correlated with admixture. Fully admixed fish being on average 2.0 ± 0.5 cm longer at smolt and 5.0 ± 0.9 cm longer after 7 months in the sea than nonadmixed fish (Figure 2f).

**FIGURE 3:**
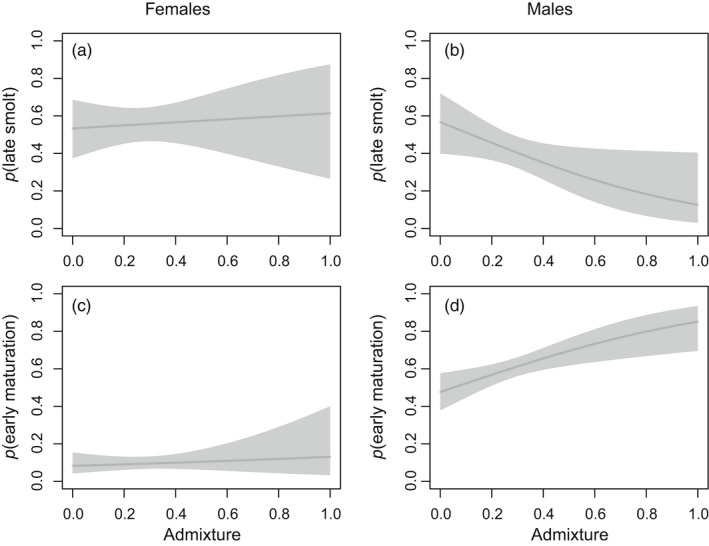
Variation in probability of late (3+ year) smolt migration for females (*p*(late smolt)) (a) and males (b), and probability of early (one sea winter) maturation for females (*p*(early maturation)) (c) and males (d) in relation to admixture from domestic strain. Regression lines are the invert logit values of the linear predictors from Generalized Linear Model (GLM) with logit link function. The shadow area on both sides of the curves represents the 95% confidence intervals

The large majority (99%) of the fish smoltified at an age of 2 or 3 years. Smolt age was thus modeled as a binary trait, and the probability of 3‐year‐old smolt was evaluated against admixture (Figure 3a,b). Smolt age and admixture were not correlated for females (Figure 3a), but there was a significant negative correlation for males (*df* = 1, *χ* ^2^ = 7.2, *p* = 7.1 × 10^−3^) (Figure 3b), with nonadmixed males migrating mainly as 3‐year‐old smolts (57%), whereas the fully admixed males migrated mainly as 2‐year‐old smolts (89%).

Age at adult maturation was also correlated with admixture for males but not in females (Figure 3c,d). For males (Figure 3d), the probability of maturing as 1 sea winter fish increased significantly with admixture (*df* = 1, *χ* ^2^ = 12, *p* = 5.5 × 10^−4^), whereas the probability of maturing as 2 or 3 sea winter fish decreased along the same gradient. Admixture was thus associated with decreasing age at maturity in the male group.

## 4 DISCUSSION

This study documents the life history and phenological characteristics of a wild Atlantic salmon population that has been subjected to long‐term introgression and admixture from nonlocal conspecifics that are both domesticated and have been subjected to directional selection for traits of importance in aquaculture for up to 15 generations. Based upon the analysis of this population, it was possible to demonstrate that admixture results in an increase in smolt size and size at maturation, a decrease in age of adult male maturation, a decrease in male smolt age, and finally, a delay in the relative timing (i.e., date upon capture in the trap) of adult migration back to the river to spawn. As the collective experimental attributes of this system are novel, these results provide an important expansion in our understanding of the consequences of gene flow from domesticated Atlantic salmon in wild populations (Glover et al., 2017). Furthermore, they contribute to our understanding of the challenges seen in other species of the Salmonidae family, which are repeatedly subjected to introgression and admixture from deliberately or accidently released nonlocal and possibly domesticated conspecifics from hatcheries (Araki et al., 2008; Laikre et al., 2010; Naish et al., 2007).

With an increase in admixture from domesticated conspecifics, we observed an increase in size at age in the sea and upon return to the river, an increase in smolt size, and a decrease in male smolt age. These observations are consistent with the fact that growth represents the trait most intensely selected for in domesticated salmon strains for aquaculture (Gjedrem, 2000, 2010) and that the offspring of domesticated Atlantic salmon outgrow wild conspecifics when reared together in farming tanks (Glover et al., 2009; Solberg et al., 2013), as well as when released into the wild (Skaala et al., 2012, 2019). These results are also consistent with the recent study of 105 Norwegian rivers (Bolstad et al., 2021), which also reported a correlation between decreased smolt age and admixture, despite a high inter‐river variation. However, the same study reported little or no effect of admixture on smolt length (Bolstad et al., 2021).

Increased size upon maturation with admixture has also been reported across multiple Atlantic salmon populations (Bolstad et al., 2017, 2021). All of these traits are linked to fast growth rate, which is selected for in domesticated Atlantic salmon. The magnitude of influence of admixture on size at maturation reported here was comparable with estimations across multiple populations (Bolstad et al., 2017, 2021) and comparable to data originating from controlled release experiments in the wild (Jonsson & Jonsson, 2017; Skaala et al., 2019). Noteworthy is the fact that in all of these studies, the relative growth differences between domesticated vs. wild Atlantic salmon, or strongly admixed vs. nonadmixed salmon, are less, and in some instances far less, in the wild than observed for the same groups under communal farming conditions (Glover et al., 2009; Solberg et al., 2013). The hypothesis suggested to explain this contrast, at least for the freshwater stage of the life cycle, is a combination of energy budget plasticity and growth‐potential selection (Glover et al., 2018). That is, a river is unlikely to provide enough energy available in prey items and/or the domesticated salmon use too much energy capturing food for them to realize their genetic growth potential. Simultaneously, there is evidence of selection against the fastest growing and highest risk‐taking domesticated fish in the river, which thus also serves to reduce the observed growth rate of the survivors (Glover et al., 2018). This hypothesis is supported by a recent study demonstrating that susceptibility to predation (Solberg et al., 2020) explains at least part of the substantial survival differences between domesticated and wild Atlantic salmon in freshwater (Fleming et al., 2000; McGinnity et al., 2003; Skaala et al., 2019; Sylvester et al., 2019).

Two earlier studies based on 62 and 105 Atlantic populations, respectively (Bolstad et al., 2017, 2021) indicated a high variability of the effect of admixture on sea age, with results showing that admixture may lead to both increases and decreases in age at maturation of males and females, depending on the genetic background of the recipient wild population. Our observation of a male‐specific decrease in age at maturation in the river Etne raises two points for discussion. First, a decrease in age at maturation with increasing admixture may appear as paradoxical since domesticated Atlantic salmon are selected against early maturation as the part of many breeding programs (Gjedrem, 2000, 2010). However, early maturation is also strongly correlated with fast growth (Gjerde et al., 1994; Taranger et al., 2010), suggesting that selection for both late maturation and fast growth is potentially incompatible. Given the well‐documented gains in growth of domesticated Atlantic salmon through selection (Debes & Hutchings, 2014; Glover et al., 2009; Solberg et al., 2013), and the fact that daylight manipulation has been used to control early maturation in commercial farming (Taranger et al., 2010), it is possible that selection for increased growth rate has directly or indirectly influenced age at maturation of the admixed males observed in the present data. The second point of interest is the observation that admixture was only correlated with male maturation age. Age at maturation in Atlantic salmon is to a degree controlled by the *vgll3* gene (Ayllon et al., 2015; Barson et al., 2015). Furthermore, sex‐dependent dominance of this gene's influence on age at maturation is believed to help maintain the differences between the sexes in their different optimal reproductive strategies (Fleming, 1996). Although not specifically tested here, it is possible that this, together with differences in underlying genomic architecture of domesticated salmon, may give rise to the sex‐dependent result. The hypothesis of a major‐effect locus being associated with the effect of introgression on life history and growth was, however, tested in a study based on 105 Norwegian rivers (Bolstad et al., 2021). This reported little or no evidence of locus effect on the effect of admixture. Similarly, the allelic frequencies of the populations at the tested loci were very close to those of the domestic strains.

Admixture delayed the date of arrival in the fish trap by up to 26 days for strongly admixed fish. Adult run timing has an underlying genetic basis in salmonids, ranging from a single gene of major influence in some Pacific salmonids (Hess et al., 2016; Prince et al., 2017; Thompson et al., 2019), to a more modest single‐locus effect in Atlantic salmon (Cauwelier et al., 2018). The mechanisms underpinning our observation, and the potential evolutionary consequences of this observation, are not immediately obvious. For example, a moderate variation in ascendance date is not expected to confer any clear advantage given that the river Etne is short enough for ascending salmon to reach spawning grounds within a day or two. However, based upon both the analysis of salmon entering the upstream trap in this river and the catch of salmon by anglers above the trap, an earlier study concluded that the timing of the angling season is likely to represent the largest directional driver of angling‐induced selection on the population in the river Etne (Harvey et al., 2017). Therefore, if migration date is considered in isolation from any other admixture‐related traits, the consequences of this are that angling has the potential to target less‐admixed salmon in the population as they enter the river earlier and are thus exposed to angling for a greater duration. Admixture would thus lead to maladaptive phenotypes in the early life stages as documented here and in many earlier studies but could be positively selected for in the later stage due to the influence of an additional anthropogenic factor, that is, angling. It is, however, not straightforward to disentangle the effect of migration date from other factors. Additionally, the willingness to bite a fishing lure, for example, may differ between late and early migrating salmon. As a result, fish entering the river prior to the fishing season are potentially less likely to bite a lure than fish entering the river during fishing season, which could compensate for the lesser angling pressure induced by delayed migration in the admixed salmon. While it is too early for management authorities to act upon this novel result, it should nevertheless be held in mind when future changes in the timing of the angling season are implemented in this, and possibly other admixed populations.

It also seems important to question the cause of the late return observed among the admixed salmon. As indicated above, adult migration timing has a genetic component in salmonids, although less well understood in Atlantic salmon compared with its Pacific counterparts. Domesticated Atlantic salmon have been directionally selected for multiple traits (Gjedrem, 2000, 2010), have their timing of maturation tuned by various environmental manipulations (Taranger et al., 2010), and not least, have been selected in the farming environment where there has been no natural selection on freshwater return or its timing, nor sexual selection, for at least 15 generations. In addition, long‐term data from Norway may suggest a decline in propensity of escaped domesticated Atlantic salmon to migrate to freshwater over time (Diserud, Fiske, et al., 2019; Glover et al., 2019). One of the possible explanations that has been put forward to explain that observation is that domestication and directional selection for other traits may have altered the migration behavior of domestic Atlantic salmon (Glover et al., 2019). That suggestion is consistent with the observations here.

Our results showed that the magnitude of change for most of the admixture‐influenced traits was modest. This observation is consistent with the results of similar previous studies (Bolstad et al., 2017, 2021; Skaala et al., 2019). The results of modeling the effect of admixture on life history traits of wild Atlantic salmon populations following introgression from domesticated and directionally selected conspecifics also indicate that under low to modest intrusion scenarios, the mean population change is likely to be weak and potentially undetectable (Castellani et al., 2015, 2018). To illustrate this point further, at 20% individual admixture, as is approximately the case for the river Etne's mean level of admixture, most traits studied here changed moderately, with for example +200 g of adult weight, or river ascension delayed by 5 days (Figure 2). As these effects were observed in the natural environment, it is possible that additional factors could affect the measured effect of admixture. Selective mortality, for example, could remove the most extreme domestic phenotypes from the population before our observation and measurement during adult migration, attenuating thus the measured difference between wild and admixed fish.

However, as suggested from the interpretation of eco genetic model results (Castellani et al., 2018), small to modest changes in population means are likely to be caused by a combination of selection removing many of the strongly admixed fish from the population in the early life history stages, which can therefore not contribute the population average in traits measured at adult stages, and that many traits in fish are highly plastic. Norway is the country displaying the greatest footprint from introgression of domesticated Atlantic salmon conspecifics in wild populations (Glover et al., 2017). A recent analysis shows that over two thirds of >200 populations investigated display detectable introgression from farmed escapees (Diserud et al., 2019). Furthermore, risk assessments have projected that this trend is set to continue in the foreseeable future (Glover et al., 2020). Nevertheless, few wild populations in Norway display mean introgression and admixture levels greater than the population studied here. This suggests that only modest phenotypic changes are likely to be seen in these populations. This is a fact that fishery managers need to bear in mind, as it serves to calibrate expectations for the effects of introgression and admixture. That is, we are not necessarily expecting to see major phenotypic changes in most robust wild populations following low to modest levels of spawning intrusion from domesticated escapees from fish farms. However, it is equally important to point out that this does not suggest that these changes are of little importance. This is because these traits are still maladaptive and are likely to undermine the genetic integrity and future evolutionary potential of these populations. Furthermore, at higher intrusion levels, these changes will manifest themselves more clearly.

## 5 CONCLUSIONS

To conclude, the present study both complements results from the previous studies of wild Atlantic salmon that have been admixed with domesticated salmon escaping from commercial fish farms, and in addition, provides knowledge of new genetic changes that may occur in wild populations. Specifically, the observation of delayed return to freshwater represents a novel result that is difficult to accurately estimate in populations without a trapping system and sheds yet further light on the potential complexities surrounding this general topic.

## CONFLICT OF INTEREST

The authors declare no conflict of interest.

## Supporting information

Figure S1

## Data Availability

Data for this study are available from the IMR.brage.unit.no public repository. Link to data: https://imr.brage.unit.no/imr‐xmlui/handle/11250/2987789.
