# Supplementary figures and images for: Introgression of domesticated salmon changes life history and phenology of a wild salmon population
Source: Evol Appl. 2022 Apr 11;15(5):853–64. doi: 10.1111/eva.13375 (PMC9108307; doi:10.1111/eva.13375)

Discriminant axis 1 (43%)

Discriminant axis 2 (16%)

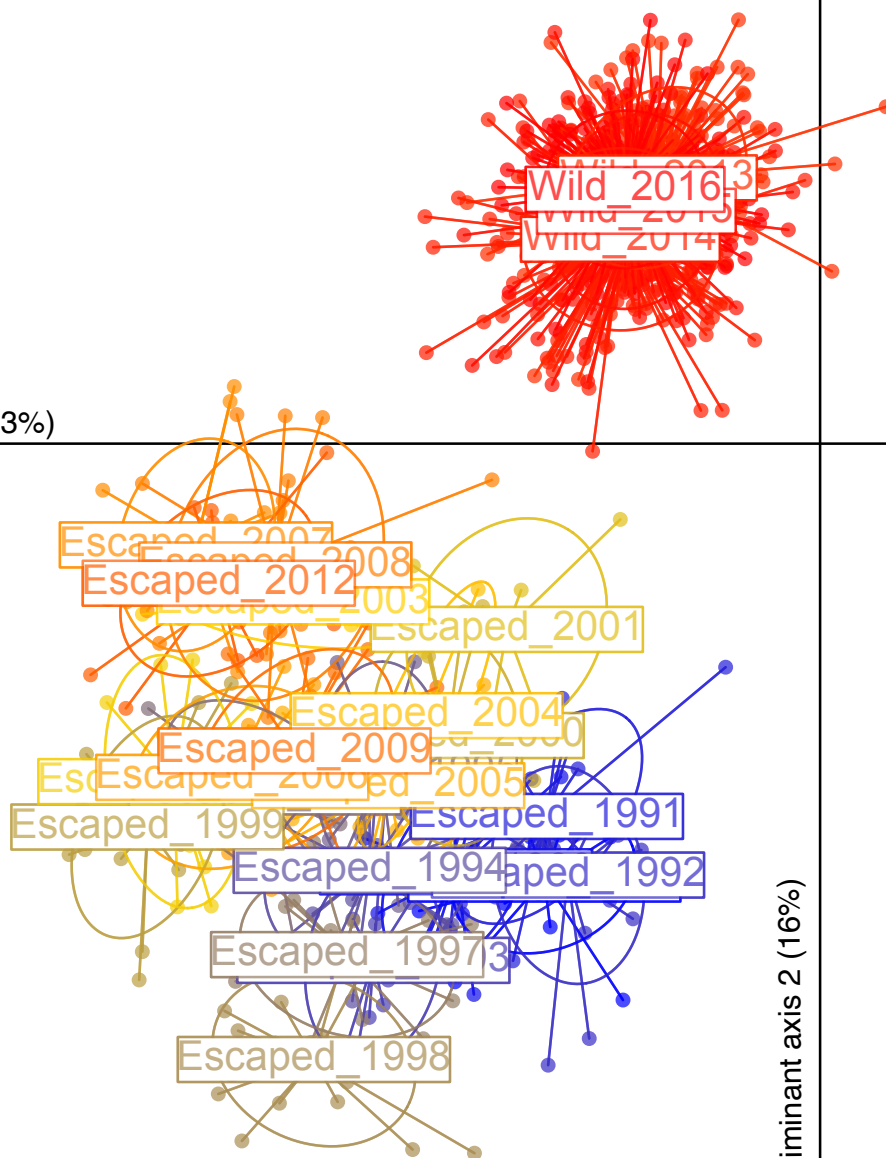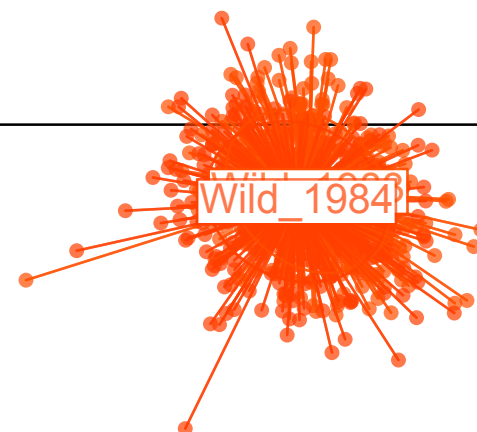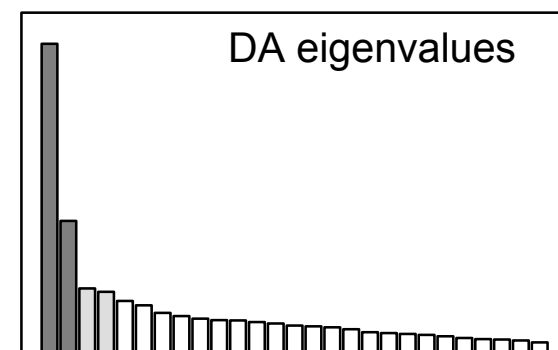

Supplement: Supplementary file 1 — Figure S1 [file EVA-15-853-s001.pdf]
